# P-518. Respiratory Tract Infections and Co-Infections Following Administration of the Respiratory Syncytial Virus (RSV) Antibody Clesrovimab versus Placebo: Analysis of the Phase 2b/3 CLEVER Trial

**DOI:** 10.1093/ofid/ofaf695.733

**Published:** 2026-01-11

**Authors:** Andrea Guerra, Ying Zhang, Radha A Railkar, Jeannine Lutkiewicz, Anushua Sinha

**Affiliations:** MSD (UK) Limited, London, UK, London, England, United Kingdom; Merck & Co., Inc., Rahway, NJ, USA, Rahway, New Jersey; Merck & Co., Inc., Rahway, NJ, USA, Rahway, New Jersey; Merck & Co., Inc., Rahway, New Jersey; Merck & Co., Inc., Rahway, NJ, USA, Rahway, New Jersey

## Abstract

**Background:**

In the phase 2b/3 CLEVER trial (MK-1654-004; NCT04767373), clesrovimab, an investigational long-acting human monoclonal antibody, reduced the incidence of RSV-associated medically attended lower respiratory tract infection (MALRI) and RSV-associated hospitalization in healthy infants, compared with placebo. RSV coinfection with other respiratory pathogens frequently occurs and may lead to increased disease burden. This exploratory analysis aimed to describe the occurrence of coinfections in the CLEVER trial.

**Methods:**

Healthy preterm and full-term infants entering their first RSV season were randomized 2:1 to receive 1 dose of clesrovimab 105 mg or placebo. Community respiratory infections were identified from nasopharyngeal samples collected during protocol-defined active surveillance for respiratory infections, from days 1-180 post dose. Pathogens included adenoviruses, human metapneumovirus, parainfluenza viruses, rhinovirus/enterovirus, influenza strains, and SARS-CoV-2.

**Results:**

This analysis consisted of participants who received clesrovimab (n = 2398) and placebo (n = 1201). Among participants with RSV-associated MALRI from days 1-180 post dose (clesrovimab: n = 64; placebo: n = 77), the majority had no coinfection (clesrovimab, 59.1%; placebo, 54.5%). The same proportion of participants (36.4%) in the clesrovimab and placebo groups had coinfection with 1 pathogen, with human rhinovirus/enterovirus being most common (clesrovimab, 30.3%; placebo, 33.8%). Coinfection with ≥ 2 pathogens was present in 9.1% of cases in the placebo group and 1.5% of cases in the clesrovimab group. The incidence of non-RSV infections was generally comparable between intervention groups with the most common being human rhinovirus/enterovirus (clesrovimab, 41.7%; placebo, 42%) and SARS-CoV-2 (clesrovimab, 12.8%; placebo, 12.1%).

**Conclusion:**

Community respiratory infections were frequently detected during active surveillance in the CLEVER trial. Coinfection was common in RSV-associated MALRI, with human rhinovirus/enterovirus isolated in > 30% of infants meeting the case definition.

**Disclosures:**

Andrea Guerra, MBBS, MSD (UK) Limited, London, UK: Employment Ying Zhang, PhD, Merck & Co., Inc., Rahway, NJ, USA: Employment|Merck & Co., Inc., Rahway, NJ, USA: Stocks/Bonds (Public Company) Radha A. Railkar, PhD, Merck & Co., Inc., Rahway, NJ, USA: Employment|Merck & Co., Inc., Rahway, NJ, USA: Stocks/Bonds (Public Company) Jeannine Lutkiewicz, BS, Merck & Co., Inc., Rahway, NJ, USA: Employment|Merck & Co., Inc., Rahway, NJ, USA: Stocks/Bonds (Public Company) Anushua Sinha, MD, Merck & Co., Inc., Rahway, NJ, USA: Employment|Merck & Co., Inc., Rahway, NJ, USA: Stocks/Bonds (Public Company)

